# Characterization and Expression Analysis of Four Cadmium-Tolerance-Associated Genes of *Avicennia marina* (Forsk.)

**DOI:** 10.3390/biology12020216

**Published:** 2023-01-30

**Authors:** Jinfeng Yu, Jicheng Zhang, Hualong Hong

**Affiliations:** 1 Key Laboratory of the Ministry of Education for Coastal and Wetland Ecosystems, School of Life Sciences, Xiamen University, Xiamen 361102, China; 2 Xiamen Innovax Biotech, Xiamen 361022, China; 3 Frasergen, Wuhan 430075, China

**Keywords:** mangrove, trace metal, peroxidase, phytosulfokine, pectate lyase, real-time quantitative PCR

## Abstract

**Simple Summary:**

The present study initially identified four full-length genes (peroxidase1, *AmPRX1*; peroxidase2 *AmPRX2*; pectate lyase, *AmPL*; phytosulfokine, *AmPSK*) associated with metal tolerance in *Avicennia marina*, one of the pioneer mangrove species with adaptive features. These genes showed the characteristic features of the respective protein families, indicating their evolutionarily conserved relationship with other plant proteins (e.g., CrPRX in *Catharanthus roseus*, MtPRX in *Medicago truncatula*, and NnPSK in *Nelumbo nucifera*). Real-time quantitative PCR revealed that *AmPRX1*, *AmPRX2*, and *AmPL* were most abundantly expressed in the leaves, while *AmPSK* displayed similar transcript levels across leaves, stems, and roots. Additionally, increased expression levels of these genes were detected in *A. marina* leaves under cadmium stress. The study thus suggests the role of these four genes in the cadmium stress response and provides a basis for detailed functional studies.

**Abstract:**

Mangroves colonize the intertidal area of estuaries (e.g., Pichavaram, Payardia, and Mai Po) with remarkable cadmium (Cd) pollution. A study on the mechanism of mangrove plant response to Cd pollution can help to understand the adaptive characteristics of plants under Cd stress. This study explored the roles of peroxidase (PRX), pectate lyase (PL), and phytosulfokine (PSK) genes in cadmium tolerance of mangrove *Avicennia marina*. Full-length sequences of four genes (i.e., *AmPRX1*, *AmPRX2*, *AmPL*, and *AmPSK*) associated with metal tolerance were identified with suppression subtractive hybridization and rapid amplification of cDNA ends. These genes showed the characteristic features of the respective protein family, indicating functions similar to other plant proteins. Real-time quantitative PCR analysis demonstrated that cadmium exposure resulted in differences in expression patterns among the tissues. Our findings emphasize the complex regulatory mechanism of these four genes in response to trace metal pollution and reveal their functions in metabolic signaling during the stress response.

## 1. Introduction

Mangroves are evergreen plant communities consisting of shrubs or trees commonly found on the coastline of tropical, subtropical, and temperate regions. They are well known for their various ecological services and socio-economic benefits [1,2]. The mangrove wetlands serve as a natural habitat for several species and act as an important resource for timber, fish, and medicinal herbs [3,4]. Mangroves also bind the soil particles together, reduce erosion, and protect the coastal regions. Mangrove sediments are known to effectively trap trace metals due to the large proportion of fine particles, high organic content, and low pH [5]. Consequently, mangroves possess an excellent tolerance for trace metal pollution [6,7]. However, more heavy metals have been released into the ecosystem via increasing anthropogenic activities, such as industrial emissions, agricultural drainage, and urban runoff [8,9,10], threatening mangrove wetlands [11]. Cadmium (Cd) is one of the most persistent and mobile heavy metals in nature and is toxic to living cells at very low concentrations [12,13]. Mangrove sediments usually contain high levels of Cd [14,15], and some mangrove plants have been found to have a high tolerance to Cd stress [16,17]. Therefore, understanding the expression pattern of genes under Cd stress can help to improve the understanding of evolved adaptive features of plants.

*Avicennia marina* (Forsk.) Vierh, commonly known as the white or grey mangrove, is a facultative halophyte [18] and one of the pioneer mangroves that have evolved to thrive under high salinity. *A. marina* exhibits greater metal accumulative and tolerance properties than other mangrove species [19]. *A. marina* has evolved specific salt-resistant features, such as salt glands and salt stomata, which help secrete excess salt to alleviate the salt-induced osmotic stress in the cells [20]; they can also excrete metals via the trichomes in the leaves. Herein, we hypothesize that such physical adaptations of *A. marina* might play a key role in maintaining homeostasis and detoxifying heavy metals in leaves [21].

Plants have evolved complex molecular mechanisms to regulate trace metals’ uptake, accumulation, transport, and detoxification [7,22]. These molecular mechanisms respond to trace metal stress at multiple stages. Firstly, plants develop adaptive regulation on their transporters, such as CDF, NRAMP, ATPases, and ZIP families [23,24], to balance the uptake and secretion of trace metals and maintain intracellular homeostasis. Once the trace metals enter the system, cysteine-rich peptides such as metallothioneins and phytochelatins are employed to chelate and then detoxify trace metals [25,26,27]. Components of the antioxidant system are the last chance for plant cells to survive under trace metal stress. These components include superoxide dismutase, peroxidase [28,29], glutathione peroxidase, and glutathione reductase [30], which keep the cellular reactive oxygen species (ROS) under control and help the cell response to trace metal stress [31,32,33].

Signaling pathways could also play a deeper role in the response to specific stress. For instance, recent studies have associated phytosulfokine (PSK) and its receptor with the abiotic stress-related signal transduction pathway in various plant species under osmotic stress and drought. However, no study has analyzed the role of PSK in metal tolerance. The partial sequencing of anonymous cDNA clones (expressed sequence tags, ESTs) is a rapid and cost-effective method for generating data on the coding regions of genomes. The abundance of such sequence information presents opportunities to accelerate progress towards understanding genetic mechanisms that control plant growth and responses to the environment [34]. Our previous study screened and identified the genes associated with trace metal tolerance in typical mangrove *A. marina* using the suppression subtractive hybridization approach [35]. However, there is still a lack of understanding of the evolutionary characteristics and expression patterns of these genes, especially with regard to comparisons with other homologous genes. The full-length cDNA clones of four selected genes related to metal tolerance were obtained by 5′ and 3′ RACE and further characterized. Their gene expression levels in the leaves, stems, and roots of *A. marina* plants exposed to cadmium stress were analyzed via quantitative real-time PCR (qPCR). This study will improve our understanding of the stress response mechanisms in mangroves.

## 2. Materials and Methods

### 2.1. Plant Materials and Cadmium Treatment

Hypocotyls of *A. marina* were collected in September 2012 from the mangrove swamps of Yunxiao, China (23°55′ N, 117°26′ E). These hypocotyls were grown in water-washed sand-filled pots [36] in the greenhouse (25 ± 5 °C, 60–80% relative humidity, and 12 h light/dark cycle at 800–1400 μmol photons m^−2^ s^−1^). The hypocotyls were irrigated with half-strength Hoagland’s solution [37] containing 10‰ sodium chloride: 5.0 mM KNO_3_, 7.0 mM Ca(NO_3_)_2_, 2 mM MgSO_4_, 2 mM KH_2_PO_4_, 26 µM Fe-EDTA, 45 µM H_3_BO_3_, 0.4 M CuSO_4_, 0.7 µM ZnSO_4_, 9.1 µM MnCl_2_, 28 mM FeSO_4_, and 0.1 µM (NH_4_)_6_Mo_7_O_24_; pH adjusted to 6.0 with 1 M NaOH. Hoagland’s solution was replaced every 3 days.

After 9 months, the hypocotyls were transferred onto a net and exposed to cadmium stress by submerging in half-strength Hoagland’s solution containing 10‰ NaCl and 2 mg L^−1^ cadmium chloride (CdCl_2_) for 1, 3, and 7 days. The Cd level was set to simulate the Cd stress in mangrove wetlands under pollution [38]. Plants submerged in half-strength Hoagland’s solution without CdCl_2_ under the same conditions as treatment were maintained as the control group. Plants were harvested after 1, 3, and 7-day exposure and washed thoroughly with deionized water. Then plant tissues were stored at −80 °C before analysis.

### 2.2. Identification of the Full-Length cDNA Sequences

Total RNA was extracted from samples following the CTAB method [39]. RNase-free DNase I (TaKaRa, Tokyo, Japan) was added to the CTAB extract to remove genomic DNA. After removing the genomic DNA, the RNA purity and integrity were assessed based on the A260/A280 ratio using Nanodrop and agarose gel (1.0%) electrophoresis. Then, 1 µg of the total RNA was used to synthesize the single-stranded cDNA using SMARTer™ PCR cDNA Synthesis Kit (Clontech, Mountain View, CA, USA).

The 5′ and 3′ end cDNA sequences of *AmPRX1*, *AmPRX2*, *AmPL*, and *AmPSK* were obtained via rapid amplification of cDNA ends (RACE) performed using the total RNA extracted from leaves of *A. marina* with the SMARTer^TM^ RACE Kit (Clontech, Mountain View, CA, USA). The first round of PCR was performed using the gene-specific primer (GSP) (Table S1) and the universal primer mix (UPM), and a second nested PCR was performed with the product of the first PCR using a nested gene-specific primer (NGSP) (Table S1) and the nested universal primer A (NUP). The final PCR products were analyzed on agarose gels, cloned into the pMD-18T vector (Takara, Tokyo, Japan), and sequenced. Finally, the 5′ and 3′ end sequences were assembled with an overlapping fragment to obtain the full-length cDNA sequences using Lasergene 7 (DNASTAR, WI, USA).

### 2.3. Feature Prediction of Deduced Proteins

The theoretical isoelectric point (pI) and the molecular weight (MW) of the deduced proteins (*AmPRX1*, *AmPRX2*, *AmPL*, and *AmPSK*) were analyzed in Lasergene 7 (DNASTAR, WI, USA). A homology search was conducted with NCBI BLAST (http://blast.ncbi.nlm.nih.gov/Blast.cgi, accessed on 1 December 2022). The three-dimensional model of each protein was built by the SWISS-MODEL server (http://swissmodel.expasy.org, accessed on 1 December 2022). The subcellular localization was predicted using PSORT (http://psort.hgc.jp/form.html, accessed on 1 December 2022). Multiple alignments of amino acid sequences homologous to *AmPRX1*, *AmPRX2*, *AmPL*, and *AmPSK* were performed using T-Coffee (https://tcoffee.org, accessed on 1 December 2022) [40] to compare the evolution of these genes. Finally, a phylogenetic tree based on amino acid sequences was constructed in Simple Phylogeny. Phylogenetic analysis of the PRX, PL, and PSK proteins was conducted based on the similarity of amino acid sequences. Four amino acid sequences in this study and isozyme sequences from GenBank were combined for phylogenetic analysis.

### 2.4. Quantitative Reverse Transcription Real-Time PCR (RT-qPCR)

Furthermore, RT-qPCR was performed to assess the mRNA expression levels of the genes associated with trace metal tolerance (Figure S1). The cDNA was synthesized using a PrimeScript^TM^ RT reagent Kit with gDNA Eraser (TaKaRa, Tokyo, Japan), and primers for the RT-qPCR were designed using Primer 5.0 (PREMIER Biosoft, San Francisco, CA, USA). Primers (Table S2) for RT-qPCR were 20–23 bp in length with melting temperatures of 55–58 °C. The PCR products were 100–120 bp in length. The *A. marina* 18S rRNA gene was used as an internal control. The RT-qPCR was carried out in a reaction mixture (18 μL) containing 12 μL of FastStart Universal SYBR Green Master (ROX; Roche Applied Science, Indianapolis, IN, USA, 2014), 0.25 μL (10 μM) each of sense and antisense primers, 2 μL of cDNA, and 3.5 μL of nuclease-free water. Reactions with cDNA replaced by nuclease-free water were run with each primer pair as a blank control. Three technical replicates were maintained per reaction, and PCR for each EST was performed in triplicate. The PCR was carried out on an ABI 7500 real-time PCR system (Applied Biosystems, Pleasanton, CA, USA) using the following program: 2 min at 50 °C, 10 min denaturation at 95 °C, followed by 40 cycles of 10 s at 95 °C and 1 min at 60 °C; finally, a melting curve analysis was performed for 30 min from 65 °C to 95 °C. The relative gene expression levels were quantified using the 2^−△△Ct^ method [41] and expressed as mean values of triplicates relative to the mean values of control at 0 days.

### 2.5. Statistical Analysis

Three replicates were involved in each treatment. Statistical analysis of data was conducted using Statistical Package for Social Science software (16.0, IBM, New York, NY, USA) and R (4.1.3). One-way analysis of variance (ANOVA) was applied to compare gene expression in different organs; two-way ANOVA was applied to the analysis of temporal expression patterns. Duncan’s multiple range test was performed to measure the differences between pairs of means. Differences in values were considered statistically significant at *p* < 0.05 unless otherwise stated.

## 3. Results

### 3.1. Structural Characterization of AmPRX1, AmPRX2, AmPL, and AmPSK cDNA Clones

**Figure 1:**
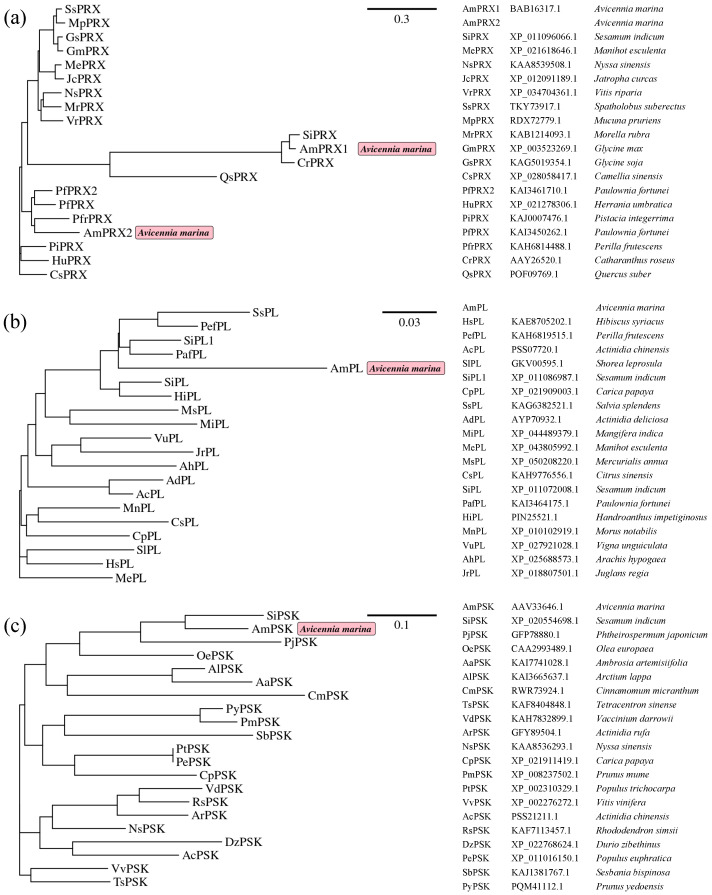
Phylogenetic tree generated using the (**a**) PRX, (**b**) PL, and (**c**) PSK proteins of various plant species. Abbreviations of 49 plant species and the GenBank accession numbers for each protein are shown in brackets.

The characteristics of the four genes associated with trace metal tolerance in *A. marina* are presented in Table 1. Phylogenetic analysis showed that the PRX proteins clustered in two major clades: One included AmPRX1 and the other included AmPRX2. Poor homology was detected in the first 20–30 amino acid residues of AmPRX1 and AmPRX2 (Figure S2a,b), suggesting the presence of specific targeting peptides. The AmPRX1 protein was closely associated with SiPRX (Figure 1a, 92.42% similarity, *Sesamum indicum*, XP_011096066.1). Meanwhile, the AmPRX2 protein was close to PfPRX (Figure 1a, 73.57% similarity, *Paulownia fortunei*, KAI3450262.1). The AmPSK protein was slightly associated with the SiPSK protein (Figure 1c, 79.22% similarity, *Sesamum indicum*, XP_020554698.1).

**Figure 2:**
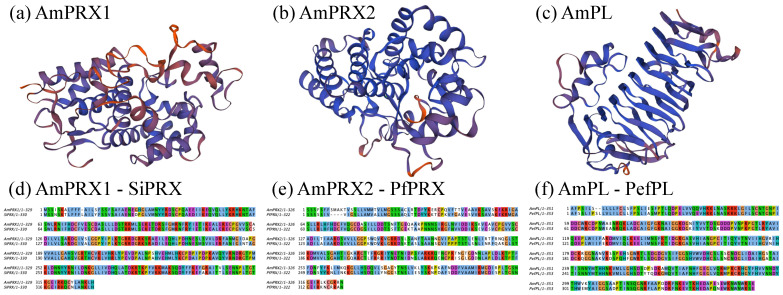
Three-dimensional models of AmPRX1 (**a**), AmPRX2 (**b**), and AmPL (**c**) proteins and alignment AmPRX1 and SiPRX (**d**), AmPRX2 and PfPRX (**e**), AmPL and PefPL (**f**). The protein models were constructed by molecular modeling in SWISS-MODEL. Color represents the estimated per-residue inaccuracy (blue and red indicate more reliable and potentially unreliable regions, respectively).

Furthermore, PSORT predicted that AmPRX1, AmPRX2, AmPL, and AmPSK are secretory proteins with signal peptides detected at their N-terminal. These genes showed the characteristic features of the respective protein family, indicating functions similar to other plant proteins (Figure 2).

### 3.2. Spatial Expression of AmPRX1, AmPRX2, AmPL, and AmPSK Genes in Avicennia marina

**Figure 3:**
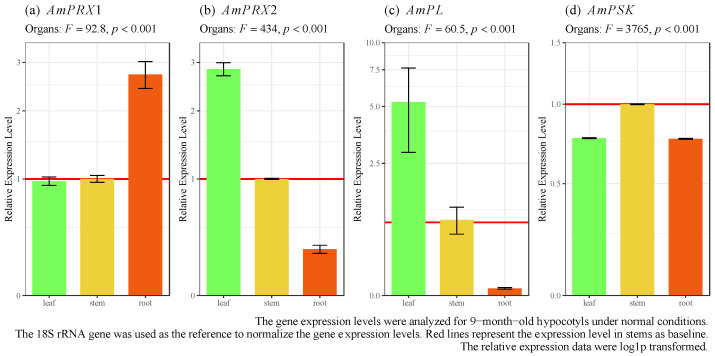
Spatial expression of *AmPRX1*, *AmPRX2*, *AmPL*, and *AmPSK* genes in *A. marina*.

Furthermore, the organ-specific expression patterns of *AmPRX1*, *AmPRX2*, *AmPL*, and *AmPSK* genes in *A. marina* under normal conditions were analyzed via RT-qPCR. These four genes were detected in all organs (leaves, stems, and roots) but with differences in expression levels (Figure 3, ANOVA, *p* < 0.05). *AmPRX1* exhibited a preference for expression in the root (Figure 3a). The expression levels of *AmPRX2* and *AmPL* were the highest in the leaves (Figure 3b,c), followed by stems and roots. Meanwhile, the *AmPSK* gene displayed comparable transcript levels among leaves, stems, and roots (Figure 3d).

### 3.3. Expression Levels of AmPRX1, AmPRX2, AmPL, and AmPSK Genes in Avicennia marina under Cadmium Stress

**Figure 4:**
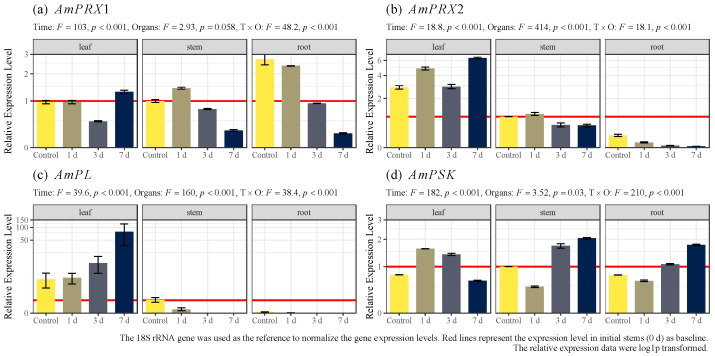
Temporal expression patterns of *AmPRX1*, *AmPRX2*, *AmPL*, and *AmPSK* genes in different tissues of *A. marina* under cadmium stress.

The temporal expression profiles of *AmPRX1*, *AmPRX2*, *AmPL*, and *AmPSK* genes in the leaves, stems, and roots of *A. marina* during seven days of cadmium exposure were determined using RT-qPCR. Cadmium stress induced the *AmPRX1* gene in the leaves on the first day (Figure 4a). Meanwhile, *AmPRX2* exhibited fluctuating expression in the leaves after cadmium exposure (Figure 4b). The expression level of *AmPL* was detectable on the first day and gradually increased until the end of the experiment (Figure 4c). The maximum expression level was detected on the seventh day, 13.5 times as many as the initial level. The expression levels of *AmPL* in the stems and roots were significantly lower than the ones in the leaves and even undetectable.

## 4. Discussion

The present study initially identified four full-length genes associated with metal tolerance (peroxidase, *AmPRX1* and *AmPRX2*; pectate lyase, *AmPL*; phytosulfokine, *AmPSK*) in *A. marina*, one of the pioneer dominant mangrove species, which might have adaptive features to metal stress. These genes showed the characteristic features of the respective protein family, indicating functions similar to other plant proteins (Figure 1 and Figure 2). RT-qPCR was used to reveal the regulation pattern of these genes in *A. marina* leaves under cadmium stress.

Peroxidase plays a crucial role in many physiological and developmental processes in plants [28,29]. The AmPRX1 and AmPRX2 proteins analyzed in this study showed a high similarity to the peroxidases of other plants. The AmPRX2 protein has a 3D structure highly similar to PePRX (Figure 2). The highly conserved structure suggests that the AmPRX2 protein might play important roles similar to other plant peroxidases. In general, the prosthetic group of peroxidase consists of protein-bound heme, which usually requires a histidine residue as a proximal ligand [44]. However, differences were detected in the N-terminal and C-terminal regions between the AmPRX1 and AtPRX2 proteins, which may be related to the adaptability of different organs of *A. marina* in response to the specific stresses in the intertidal wetlands.

The study identified AmPRX1 and AtPRX2 as secretory peroxidases with signal peptides detected at their N-terminal. These genes belong to class III of the plant heme-dependent peroxidase superfamily, which contains several conserved features, including four disulfide bridges and two calcium-binding sites [45]. Members of this class are implicated in hydrogen peroxide regulation during cell protection and signaling under abiotic stress [46]. Mangroves have an efficient antioxidant defense system. Evidence showing mangrove tissue differences in gene expression will be helpful to reveal the maintenance of cellular homeostasis in plants.

Furthermore, the RT-qPCR revealed differences in the expression levels of *AmPRX1* and *AmPRX2* among the various organs (leaves, stems, and roots) under normal conditions. *AmPRX1* and *AmPRX2* were highly expressed in the leaves of *A. marina*. For instance, *AmPRX2* showed the highest expression level among these genes, 9.1 times higher than that in the roots. These differences in expression patterns among the organs may be associated with their various physiological functions. Several researchers have reported tissue specificity of peroxidase paralogous [47], suggesting that differences in tissue expression and regulation are key parts of species evolution that need to be further explored. Specifically, a high peroxidase expression in the leaves can effectively prevent ROS-induced chloroplast damage, ensuring efficient energy acquisition and photosynthesis to encounter stress.

By regulating the hydrolysis of pectic polysaccharides, which are prominent components of plant cell walls, pectate lyase not only plays essential roles in plant growth and development but is also closely involved in signaling and defense responses [48,49,50,51]. The expression of *AmPL* was much higher in leaves than in stems and roots (Figure 3). Meanwhile, the expression of *AmPL* remained continuously up-regulated during 7 days of Cd exposure (Figure 4). This evidence suggests the specificity of *AmPL* in leaf resistance to Cd stress. Pectate lyases are depolymerizing plant enzymes involved in cell wall degradation. They catalyze the cleavage of pectate, the de-esterified product of pectin, which is the major component that maintains the structural integrity of cell walls in higher plants [52]. In *Arabidopsis*, PL-like proteins control stomatal development; they are known to cleave pectins and regulate stomatal dynamics, likely playing essential roles during metal toxicity.

Phytosulfokine, a sulfated pentapeptide growth factor, is an important part of the stimulation of plant proliferation [53] and integrates the growth and defense of plants to balance the competing metabolic costs [54]. Unlike the other three genes in this study, the expression of *AmPSK* was slightly higher in stems than in leaves and roots but did not show significant differences overall. Furthermore, the expression of *AmPSK* differed among tissues during Cd exposure. In leaves, *AmPSK* expression peaked on days 1–3, while in stems and roots, days 3–7 were the periods when *AmPSK* was primarily upregulated. We speculate that this is related to the overall regulation of *AmPSK* as a growth factor. PSK is a plant peptide growth factor with hormone-like mitogenic activity; it promotes plant growth via cellular growth and expansion in plants [55].

It must be noted that *AmPRX2* was upregulated in the leaves under cadmium stress, while *AmPRX1* and *AmPRX2* were downregulated in the stems and roots, suggesting a potential, complex role of PRX genes under cadmium stress. Considering the correlation between trace metal tolerance and the upregulation of PRX genes and the downregulation in the stems and roots, it can be assumed that multiple mechanisms are involved in trace metal tolerance in *A. marina*. In the present study, these four genes were identified in the leaves. Clearly, there are some differential Cd adaptation mechanisms in the roots and stems [38,56,57]. This also represents a wide range of adaptive features of organisms in adaptation to stress, and more refined regulatory mechanisms need to be shown in future studies with the help of spatial transcriptome tools [58].

As an adaptation to intertidal metal enrichment, mangroves develop various defense strategies to detoxify or withstand high concentrations of heavy metals (Table 2). Typically, mangroves adopt an avoidance strategy and restrict metal uptake [14,59]. Once this strategy fails, plants express specific genes to regulate the metal translocation [25,26,27,60,61,62]. Later, antioxidant substances are activated [28,29,30,31,32,33,63]. Exposure to cadmium induces oxidative stress, increasing ROS production and lipid and protein oxidation [38,64,65,66]. In the present study, cadmium exposure activated the expression of *AmPRX1*, *AmPRX2*, *AmPL*, and *AmPSK* genes in *A. marina*, especially in the leaves. Many peroxidase homologs, such as the *OsAPX* genes of *Oryza sativa*, the *BvpAPX* genes of *Beta vulgaris*, and the *LjGPX* genes of *Lotus japonicas*, have been found to play crucial roles in abiotic stress response [29,67,68].

## 5. Conclusions

This study reveals the expression patterns of four genes associated with trace metal tolerance in *A. marina*. For instance, a significant upregulation (13.5 times) of *AmPL* was detected in leaves after 7 days of cadmium exposure; however, no significant differences in *AmPL* expression were detected. Additionally, the *AmPSK* gene of *A. marina* exhibited upregulation in the leaves, stems, and roots after cadmium exposure. These observations collectively reveal the precise regulation of the metal tolerance-associated genes at the organ level. However, detailed studies are necessary to explore their roles in providing tolerance, elucidate the complex network of molecular mechanisms, and analyze the synergistic effect of multiple genes in mangrove plants under trace metal stress. Further research is necessary to understand the specific functions and regulatory mechanisms of *A. marina*, especially in the response mechanisms of stems and roots.

## Figures and Tables

**Table 1:** Characteristics of genes associated with trace metal tolerance in *Avicennia marina*.

Gene	Full Length	5′ UTR	3′ UTR	ORF	Predicted MW	Theoretical pI	Cloned by	Accession Number
	(bp)	(bp)	(bp)	(bp)	(kDa)			
*AmPRX1*	1345	48	301	996	37.60	8.41	[42]	AB049589.1
*AmPRX2*	1404	70	341	993	36.05	9.58	This study	OQ160797
*AmPL*	1545		393	1152	42.85	6.92	This study	OQ160798
*AmPSK*	727	162	331	234	55.43	5.06	[43]	AY639950.1

**Table 2:** Summary of defense strategies of mangroves associated with trace metal tolerance.

Interface	Key Adaptive Feature	Mechanism	Reference
sediment-root	rhizosphere microbiome	phytoavailability	[59]
root	anatomical structures	stress-regulated genes; metal translocation	[60]
root	gene expression; phytohormone	stress-regulated genes	[16]
root-leaf	gene expression; enzyme activity	stress-regulated genes	[63]
sediment-root	iron plaque	metal segregation	[69]
sediment-root	root exudates	phytoavailability	[70]
root	enriched substances	cellular structure	[57]
root-leaf	anatomical structures	metal translocation	[61]
leaf	enriched substances	detoxification	[38]
sediment-root	metal speciation	phytoavailability	[14]
sediment-root	root exudates	phytoavailability	[36]
leaf	gene expression; enzyme activity	stress-regulated genes; detoxification	[71]
leaf	gene expression	stress-regulated genes	[72]
sediment-root	anatomical structures	phytoavailability	[73]
root-leaf	subcellular distribution	metal translocation	[62]
leaf	gene expression	stress-regulated genes	[74]
leaf	gene expression	stress-regulated genes	[6]
stem-leaf	anatomical structures	metal translocation	[75]

## Data Availability

The data reported in this article are available in the main text or Supplementary Information, and metadata can be acquired directly from the corresponding author H.H. on request.

## Supplementary Materials

The following supporting information can be downloaded at: https://www.mdpi.com/article/10.3390/biology12020216/s1, Figure S1. Diagram for the RT-qPCR protocol; Figure S2. Multiple sequence alignment of PRX (a,b), PL (c), and PSK (d) proteins; Table S1. Primers used for RACE in this study; Table S2. Primers used for qRT-PCR analysis in this study.
