# Supplementary material for: Characterization and Expression Analysis of Four Cadmium-Tolerance-Associated Genes of Avicennia marina (Forsk.)
Source: Biology (Basel). 2023 Jan 30;12(2):216. doi: 10.3390/biology12020216 (PMC9952839; doi:10.3390/biology12020216)
Supplement: Supplementary file 1 [file biology-12-00216-s001.zip › biology-2105105-supplementary.pdf]

Characterization and Expression Analysis of Four Cadmium-Tolerance-Associated Genes of *Avicennia marina* (Forsk.)

Jinfeng Yu [1,2,†], Jicheng Zhang [1,3,†] and Hualong Hong [1,*]

[1] Key Laboratory of the Ministry of Education for Coastal and Wetland Ecosystems, School of Life Sciences, Xiamen University, Xiamen 361102, China
[2] Xiamen Innovax Biotech, Xiamen 361022, China
[3] Frasergen, Wuhan 430075, China
* Correspondence: honghl@xmu.edu.cn
† These authors contribute equally to this work.

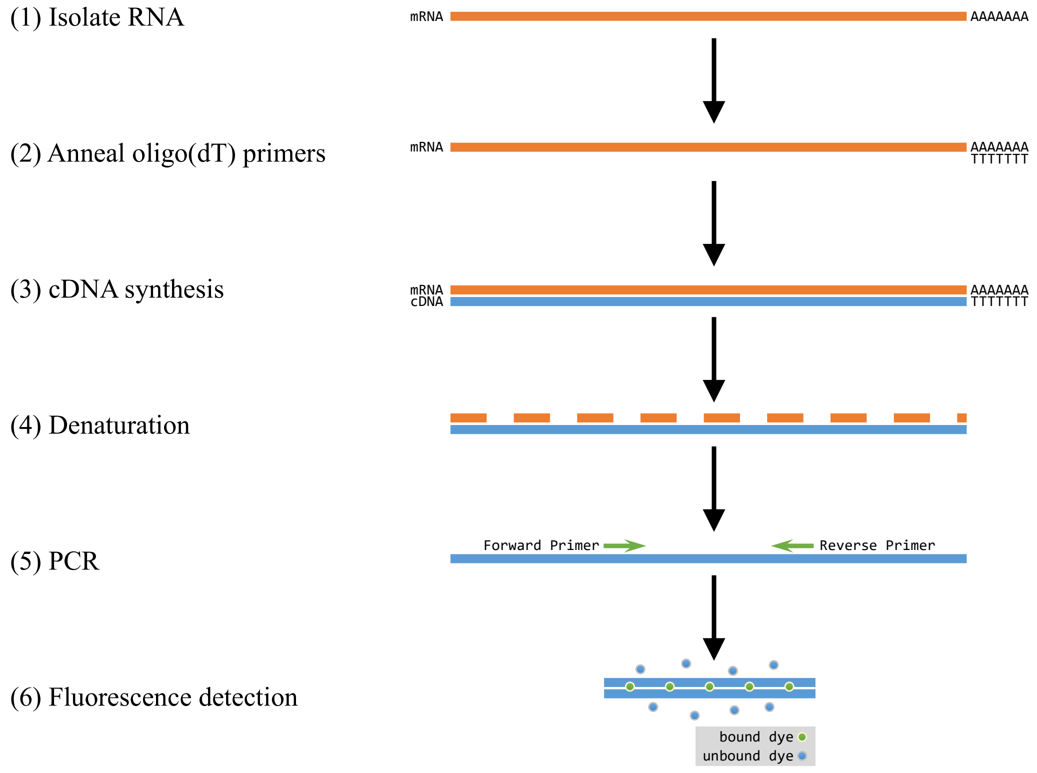

Figure S1 Diagram for the RT-qPCR protocol.

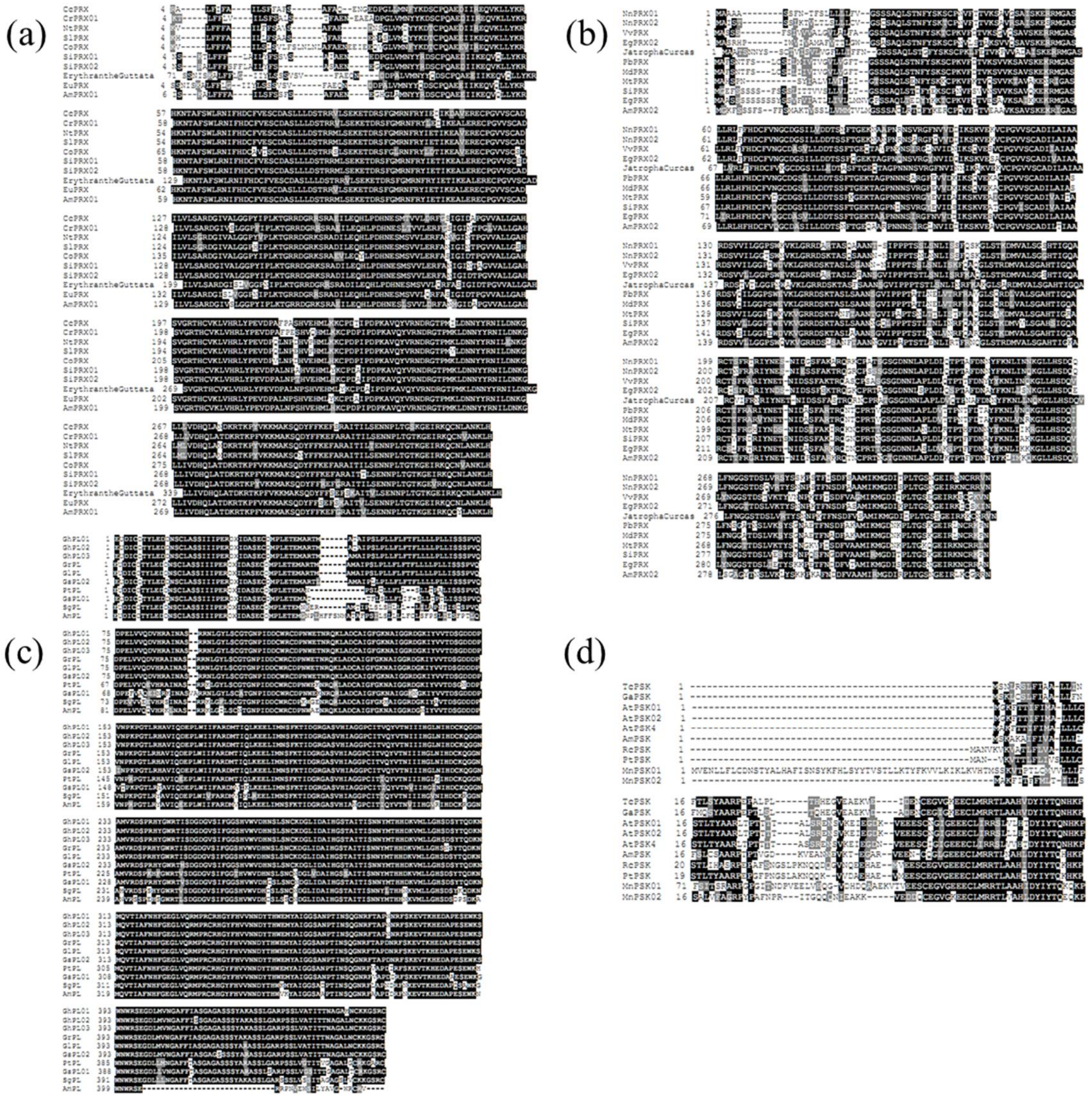

Figure S2 Multiple sequence alignment of PRX (a) (b), PL (c), and PSK (d) proteins.

The deduced amino acid sequences of PRX, PL, and PSK proteins using MEGA 5.1.

Table S1 Primers used for RACE in this study

Primers	Sequences (5'–3')
AmPL-GSP1	TGCTCTGGCGAGGACCGCACATT
AmPL-GSP2	GCAGAGAACCGCCAAAAGTTAGCCG
AmPL-NGSP1	GCATAACGGAGTGTCCCCGGCCT
AmPL-NGSP2	TGGGGACGACGACCCTGTCAACC
AmPRX2-GSP1	CGCTTCTCTCCTTCGCCTCCACTTT
AmPRX2-GSP2	GGAATCACACCGTTATTTGCCGC
AmPRX2-NGSP1	GCTTCGTTCAAGGCTGTGATGCGTC
AmPRX2-NGSP2	CGGGCAGCAATGGTTAGGATGTCG

Table S2 Primers used for qRT-PCR analysis in this study

Gene	Sequences (5'–3')	
	Forward	Reverse
Am18S	CCCGTTGCTGCGATGAT	GCTGCCTTCCTTGGATGTG
AmPRX1	AGCCACGGACAAGAGGAC	ACCCTTTGTGCCAGTGAG
AmPRX2	CAATGGTATGTGCTCCTG	TCGTTATCAGCCAACTTTA
AmPL	ACGACCCTGTCAACCCTA	CATTATCAATTCCTCCTTCA
AmPSK	TTCTCCTGCTCTTCTCCC	ACTCTGGCTCCCTCTGTC
